# Radiolabeling and PET–MRI microdosing of the experimental cancer therapeutic, MN-anti-miR10b, demonstrates delivery to metastatic lesions in a murine model of metastatic breast cancer

**DOI:** 10.1186/s12645-021-00089-5

**Published:** 2021-07-08

**Authors:** Mariane Le Fur, Alana Ross, Pamela Pantazopoulos, Nicholas Rotile, Iris Zhou, Peter Caravan, Zdravka Medarova, Byunghee Yoo

**Affiliations:** 1 MGH/MIT/HMS Athinoula A. Martinos Center for Biomedical Imaging, Department of Radiology, Massachusetts General Hospital and Harvard Medical School, Boston, MA 02129 USA; 2 Institute for Innovation in Imaging, Massachusetts General Hospital, Boston, MA 02129 USA

**Keywords:** MicroRNA, RNA interference, Metastasis, Biodistribution, Positron emission tomography, MR imaging

## Abstract

**Background:**

In our earlier work, we identified microRNA-10b (miR10b) as a master regulator of the viability of metastatic tumor cells. This knowledge allowed us to design a miR10b-targeted therapeutic consisting of an anti-miR10b antagomir conjugated to ultrasmall iron oxide nanoparticles (MN), termed MN-anti-miR10b. In mouse models of breast cancer, we demonstrated that MN-anti-miR10b caused durable regressions of established metastases with no evidence of systemic toxicity. As a first step towards translating MN-anti-miR10b for the treatment of metastatic breast cancer, we needed to determine if MN-anti-miR10b, which is so effective in mice, will also accumulate in human metastases.

**Results:**

In this study, we devised a method to efficiently radiolabel MN-anti-miR10b with Cu-64 (^64^Cu) and evaluated the pharmacokinetics and biodistribution of the radiolabeled product at two different doses: a therapeutic dose, referred to as macrodose, corresponding to ^64^Cu-MN-anti-miR10b co-injected with non-labeled MN-anti-miR10b, and a tracer-level dose of ^64^Cu-MN-anti-miR10b, referred to as microdose. In addition, we evaluated the uptake of ^64^Cu-MN-anti-miR10b by metastatic lesions using both in vivo and ex vivo positron emission tomography–magnetic resonance imaging (PET–MRI). A comparable distribution of the therapeutic was observed after administration of a microdose or macrodose. Uptake of the therapeutic by metastatic lymph nodes, lungs, and bone was also demonstrated by PET–MRI with a significantly higher PET signal than in the same organs devoid of metastatic lesions.

**Conclusion:**

Our results demonstrate that PET–MRI following a microdose injection of the agent will accurately reflect the innate biodistribution of the therapeutic. The tools developed in the present study lay the groundwork for the clinical testing of MN-anti-miR10b and other similar therapeutics in patients with cancer.

**Supplementary Information:**

The online version contains supplementary material available at 10.1186/s12645-021-00089-5.

## Background

Conventional therapies targeted towards the primary tumor cell oftentimes do not affect the metastatic cell and, in fact, may promote metastasis. This explains the poor outcomes in patients diagnosed with metastatic disease despite the good prognosis of patients with localized cancer of the same organ of origin (Steeg 2016). For these reasons, our research has focused on developing therapies specific to unique properties of metastatic tumor cells. These cells have the ability to break out of the primary tumor mass, travel through the circulation, and colonize a new vital organ in the process of metastasis. Importantly, these cells are genetically and phenotypically distinct from the majority of the cells in the tumor mass, spawning metastatic lesions that have diverged in their gene expression profile from their respective primary tumors.

In our earlier work, we showed that the metastamir microRNA-10b (miR10b) powerfully promotes the viability of metastatic tumor cells. We confirmed the earlier reports that miR10b promotes the invasion and migration of tumor cells (Ma et al. 2007, 2010). We also demonstrated that miR10b facilitates the survival of metastatic tumor cells (Yigit et al. 2013; Yoo et al. 2015, 2017b). Based on this knowledge, we developed a therapeutic strategy against metastatic cancer centered around the inhibition of miR10b in tumors and metastases. The inhibition of miR10b was achieved using antagomirs conjugated to ultrasmall iron oxide magnetic nanoparticles (MN), which served as carriers for the antagomirs. We termed this novel therapeutic MN-anti-miR10b. We demonstrated that MN-anti-miR10b could completely prevent the formation of de novo metastases (Yigit et al. 2013) and, when combined with a low-dose cytostatic, caused complete and persistent regression of local lymph node and distant metastases in breast cancer models with no evidence of systemic toxicity (Yoo et al. 2015, 2017b). Detailed molecular studies showed that the mechanism of action of MN-anti-miR10b is entirely novel and relies on a process which we have termed “metastamir addiction” (Yoo et al. 2015). Metastamir addiction refers to a property of metastatic tumor cells, according to which they upregulate the expression of specific microRNAs as an adaptive advantage. This advantage promotes the capacity of tumor cells to survive, invade surrounding tissue, and migrate in response to physiological stress caused by insufficient vascular supply, low pH, poor cell–cell contacts, and inadequate extracellular matrix (ECM) support.

From our earlier studies in animal models, we learned that the delivery of MN-anti-miR10b and other MN-based agents to tumors and metastases relies on a combination of hemodynamic, physicochemical, and metabolic factors (Arami et al. 2015; Sharma et al. 2018). They distribute to the interstitium of tumors and metastases via the enhanced permeability and retention (EPR) effect, followed by cell uptake of the positively charged nanoparticles through macropinocytosis (Juliano 2016; Medarova et al. 2007; Moore et al. 2004).

As a first step towards translating MN-anti-miR10b for the treatment of metastatic breast cancer, we needed to determine if MN-anti-miR10b, which is so effective in mice, will also accumulate in human metastases. Towards that goal, we developed a method to radiolabel MN-anti-miR10b with Cu-64 through a NODAGA chelator. The choice of Cu-64 for radiolabeling was motivated by the fact that the radionuclide has a low β^+^ energy (*E*_β+,max_ = 656 keV), which is comparable to that of ^18^F (*E*_β+,max_ = 633 keV) and would ensure a high sensitivity of detection (Williams et al. 2005). Also, Cu-64 has a 12.7-h half-life, allowing for adequate assessment of the slow pharmacokinetics of macromolecules or blood-pool agents (Williams et al. 2005; Woo et al. 2018). The NODAGA chelator was also found to be optimal because it forms a stable complex with Cu-64 and permits the accurate determination of the biodistribution of the radiolabeled entity (Desogere et al. 2015; Pretze et al. 2019; Roosenburg et al. 2014).

In the current study, a microdose of ^64^Cu-MN-anti-miR10b was injected into murine models of metastatic breast cancer. Its uptake by the metastatic lesions was determined using simultaneous positron emission tomography–magnetic resonance imaging (PET–MRI). PET–MRI clearly demonstrated uptake of ^64^Cu-MN-anti-miR10b by the metastatic lesions, measurable at a microdose. Ex vivo PET–MRI confirmed that the activity was associated with the metastatic lesions, as identified at necropsy. Uptake by metastatic organs was also demonstrated through ex vivo quantification by gamma counting and shown to be significantly higher than in the same organs devoid of metastatic lesions. Finally, we demonstrated that a microdose of ^64^Cu-MN-anti-miR10b showed the same biodistribution as a standard therapeutic dose. These preclinical studies set the stage for a clinical investigation of ^64^Cu-MN-anti-miR10b delivery to human metastases, which represents a critical step towards translating this and other similar nanotherapeutics.

## Materials and methods

### General methods

All reactants and reagents were of commercial grade and were used without further purification. All solutions were prepared from MilliQ water. Metal-free buffer solutions used for radiolabeling were prepared using Chelex 100 Resin (100–200 mesh, BioRad). All animal experiments were performed in compliance with institutional guidelines and approved by the Institutional Animal Care and Use Committee at Massachusetts General Hospital (Boston, MA).

### Synthesis of NODAGA-MN-anti-miR10b

**Scheme 1.**
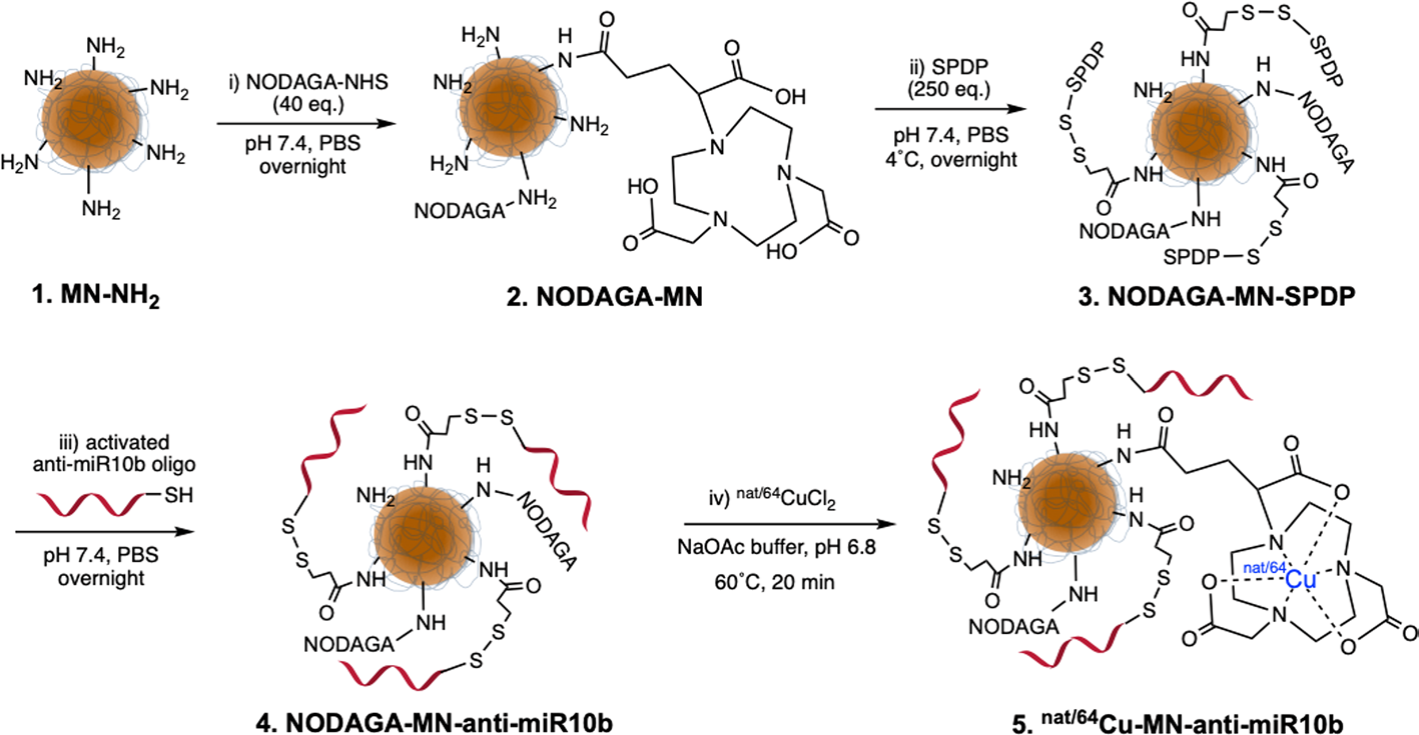
Preparation of ^nat/64^Cu-MN-anti-miR10b: **1.** Coupling reaction between MN-NH_2_ and NODAGA-NHS to form NODAGA-MN. **2.** Functionalization with the hetero-bifunctional linker, SPDP. **3.** Conjugation to anti-miR10b antagomir via disulfide linkage to form NODAGA-MN-anti-miR10b. **4.** Complexation reaction with ^nat^CuCl_2_ or ^64^CuCl_2_ leading to ^nat/64^Cu-MN-anti-miR10b

The steps for the synthesis of ^nat/64^Cu-MN-anti-miR10b are outlined in Scheme 1. Amine-derivatized iron oxide nanoparticles (MN) were prepared from dextran-coated iron oxide nanoparticles through modification with epichlorohydrin and ammonium hydroxide as described previously (Yoo et al. 2014). The nanoparticles were conjugated with NODAGA-NHS (Chematech, France) by reacting 1 ml of MN (87 μM, 10 mg Fe/ml, 54 NH_2_/MN) with 2.54 mg of NODAGA-NHS ester (3.47 μmol, 40 eq. to MN) in 100 μl PBS buffer (100 mM, pH 7.4). The reaction was carried out overnight at 4 °C, then the resulting NODAGA-conjugated nanoparticles (NODAGA-MN) were purified with a size exclusion column (PD-10, GE Healthcare) using nuclease-free PBS buffer as an eluent. NODAGA-MN was treated with excess amounts of SPDP (250 eq.) for 4 h at 4 °C to form NODAGA-MN-SPDP, which was again purified with a size exclusion column using nuclease-free PBS buffer as an eluent. The LNA antagomir, anti-miR10b is synthesized and provided by Biospring (Frankfurt, Germany) following a GLP protocol. The anti-miR10b LNA antagomir was modified with the 5′-Thiol-Modifier C6 disulfide (5′-ThioMC6), which was utilized for conjugation to MN. The disulfide on the oligonucleotide was activated by 3% Tris (2-carboxyethyl) phosphine hydrochloride (TCEP, Thermoscientific Co.), followed by purification with ammonium acetate/ethanol precipitation treatment prior to conjugation to MN. After TCEP activation and purification, the oligo was dissolved in nuclease-free water and incubated with NODAGA-MN-SPDP overnight. The final product, NODAGA-MN-anti-miR10b, was freshly prepared prior to animal studies.

### Preparation of non-radioactive ^nat^Cu-MN-anti-miR10b

Nonradioactive ^nat^Cu-MN-anti-miR10b was prepared to evaluate the inhibitory effect of miR-10b in 4T1 cells. 0.6 mg Fe of NODAGA-MN-anti-miR10b was dispersed in acetate buffer (500 µL, pH 6.8, 0.1 M) followed by the addition of CuCl_2_ (0.7 mg, 50 equiv. Cu^2+^ to NODAGA). The reaction mixture was stirred at 60 °C for 20 min and EDTA (100 µL, 100 mM, pH 7.4) was added to the mixture to remove any unlabeled free Cu^2+^ ions followed by purification with size exclusion column (PD-10, GE Healthcare) using nuclease-free PBS buffer as an eluent. Fractions containing the desired product were combined and the concentration of ^nat^Cu-MN-anti-miR10b was determined by ICP-MS.

### Preparation of radiolabeled MN-anti-miR10b (^64^Cu-MN-anti-miR10b)

Radiolabeling was performed following commonly used procedures (Désogère et al. 2017). Briefly, 200 µg (as Fe) of NODAGA-MN-anti-miR10b in PBS was added to a solution of ^64^CuCl_2_ (4 mCi, 148 MBq, the University of Wisconsin at Madison, WI) in sodium acetate buffer (0.1 M, pH 6.8, 500 µL). The reaction mixture was heated at 60 °C for 20 min and then purified with a size exclusion column (PD-10 column) using nuclease-free PBS buffer as an eluent and each 500 µL of eluent was collected as a fraction. The radiochemical purity of each fraction was controlled by iTLC (Agilent, iTLC-SG, Santa Clara, CA) with an EDTA solution as an eluent (50 mM, pH 5) using a radio-TLC imaging scanner (AR-2000, Eckert & Ziegler, Berlin, Germany). Fractions with a radiochemical purity > 99% were combined and used for in vivo animal studies. Radiochemical identity of the final solution of ^64^Cu-MN-anti-miR10b was confirmed by analytical HPLC (Agilent 1100 HPLC system, Santa Clara, CA) with a size exclusion column (TSK gel QC-PAK-300, isocratic, 100% sodium phosphate 0.1 M pH 7.4, 20 min) and a Carroll/Ramsey radioactivity detector with a silicon PIN photodiode and with UV detection at 254 nm.

### Characterization of MN-anti-miR10b and ^nat^Cu-MN-anti-miR10b

ICP-MS analysis (Agilent 8800-QQQ system, Santa Clara, CA) was carried out to determine the concentrations of copper and iron. All samples were prepared by weight. Calibration standards were prepared by diluting certified copper and iron standards (1000 mg/L). Calibration curve was obtained from 5 standard solutions in the range from 0.1 to 400 ppb. Lutetium (1 ppm) was used as an external standard to ensure the proper introduction of the sample. The hydrodynamic diameter and Zeta-potential were measured by a dynamic light scattering spectrometer (Zetasizer Nano, Malvern, UK) and the size of the iron oxide core was determined by transmission electron microscopy (JEM 2100 TEM, Jeol, Tokyo, Japan). To quantify the number of NODAGA per MN, the number of amines per MN was subtracted from the number of amines per MN after conjugation with NODAGA. The number of amines per MN was quantified by pyridine-2-thione (343 nm, 8080 M^−1^ cm^−1^) released from SPDP that was conjugated to the amine groups at a one-to-one ratio (ThermoFisher, Waltham, MA). Finally, the number of oligonucleotides per MN was determined by spectrophotometry with multiple standards of different concentrations. Briefly, MN-anti-miR10b and ^nat^Cu-MN-anti-miR10b were purified using a magnetic column (MACS column, Miltenyi, Cambridge, MA) to remove unbound anti-miR10b oligo. The purified nanoparticles were assayed to determine iron concentration (410 nm) and the concentration of oligo (260 nm) by spectrophotometry (Spectramax M2 microplate reader, Molecular Devices, Sunnyvale, CA).

### Cellular uptake

The cellular uptake of ^nat^Cu-MN-anti-miR10b was compared with that of MN-anti-miR10b and parent MN. 4T1-luc cells were seeded in a 12-well plate and incubated with ^nat^Cu-MN-anti-miR10b, MN-anti-miR10b, and MN for 24 h at 37 °C. After washing with DPBS, the cells were lysed (Cell lysis buffer, Sigma-Aldrich, St. Louis, MO) and analyzed by ICP-MS to determine the concentration of iron. The protein concentration was determined by BCA assay (Sigma-Aldrich, St. Louis, MO). The cellular uptake of nanoparticles was normalized by total protein.

### Real-time quantitative reverse transcription-PCR

To assess target engagement by ^nat^Cu-MN-anti-miR10b as compared to the unlabeled MN-anti-miR10b, 4T1-luc cells were incubated with ^nat^Cu-MN-anti-miR10b, MN-anti-miR10b, and MN for 48 h at 37 °C. From the cell lysates, the microRNA-enriched fraction was harvested using a miRNeasy mini kit following the manufacturer’s protocol (Qiagen Inc., Hilden, Germany). Relative expression of miR-10b was determined by real-time quantitative reverse transcription-PCR (qRT-PCR; Taqman protocol) and normalized to the internal housekeeping gene, SNORD44. Taqman analysis was carried out using an ABI Prism 7700 sequence detection system (Applied Biosystems, Foster City, CA). The primers (Hs-miR-10b-3 miScript Primer, Hs-SNORD44-11 miScript Primer) and assay kit (miScript PCR Starter Kit, Qiagen, Hilden, Germany).

### Animal model and administration of ^64^Cu-MN-anti-miR10b

Eight-week-old female Balb/c mice (The Jackson Laboratory; Bar Harbor, ME) were implanted orthotopically under the top right third mammary fat pad with the 4T1-Red-Fluc cell line (0.5 × 10^6^ cells). The cells express luciferase and can be detected by non-invasive bioluminescence imaging (BLI) for corresponding analysis of tumor burden. All animals were scanned by BLI to keep track of metastasis formation twice a week. Two weeks after cell inoculation, mice were injected intravenously with ^64^Cu-MN-anti-miR10b. For the microdosing studies, ^64^Cu-MN-anti-miR10b prepared as described above, was injected at a dose of 20 μg as Fe, 118–190 μCi per mouse, *n* = 6. For the carrier-added macrodosing studies, ^64^Cu-MN-anti-miR10b was mixed with NODAGA-MN-anti-miR10b and injected at a dose of 300 µg as Fe, 127–135 µCi per mouse, *n* = 7. An aliquot of the injected dose was analyzed for %ID/g calculations.

After PET–MR imaging, mice were killed at 24-h post-injection (*n* = 3 in microdose group and *n* = 4 in macrodose group) and 48-h post-injection (*n* = 3 in each group) for ex vivo biodistribution analysis.

### Bioluminescence optical imaging (BLI)

BLI was used to identify metastases. Imaging was performed using the IVIS Spectrum imaging system (Perkin Elmer, Hopkinton, MA). Anesthetized mice were injected intraperitoneally with d-luciferin potassium salt in DPBS (200 mL of 15 mg/mL; Perkin Elmer, Hopkinton, MA) 12 min before image acquisition. Identical imaging acquisition settings (time, ~ 0.5–60 s; F-stop, 2; binning, medium) and the same ROI were used to obtain total radiance (photons/sec/cm^2^/sr) over the whole body. BLI was performed for about 6 to 15 min to obtain the maximum radiance. All images were processed using the Living Image Software (ver 4.5, IVIS Spectrum, Perkin Elmer, Hopkinton, MA). The total radiance from the bioluminescence readings was used for signal quantification.

### PET–MR imaging

Mice were imaged in a 4.7-Tesla MRI scanner equipped with a PET insert (Bruker, Billerica MA). Mice were anesthetized with 1–2% isoflurane in medical air. Mice were kept warm using an air heater system and body temperature and respiration rate monitored by a physiological monitoring system (SA Instruments Inc., Stony Brook NY) throughout the imaging session. For the microdosing studies, dynamic PET acquisition was performed continuously for 1 h after injection of ^64^Cu-MN-anti-miR10b. Mice were then returned to their cages and imaged again at 2 h, 4 h, 24 h and 48 h post-injection for a period of 30 min, 30 min, 60 min, and 60 min, respectively. For the macrodosing studies, mice were scanned at 24 h after injection of ^64^Cu-MN-anti-miR10b for 60 min. For ex vivo imaging, organs were positioned onto a plastic holder and scanned for 15 min.

Anatomic MR images were obtained simultaneously with PET acquisition, including T1-weighted 3D FLASH (Fast Low Angle Shot) sequences with the following parameters: echo time (TE) = 3 ms, repetition time (TR) = 20 ms, imaging resolution = 0.25 × 0.25 × 0.5 mm^3^/voxel, and flip angle = 12°.

PET–MR imaging data were analyzed to estimate the biodistribution and clearance of ^64^Cu-MN-anti-miR10b. Regions of interest (ROIs) were drawn on the MR images over major organs, including heart, liver and kidneys using AMIDE software package (Loening and Gambhir 2003), and used for quantifying radioactivity for each PET frame. The uptake of ^64^Cu-MN-anti-miR10b in metastases and corresponding tissues without metastases was quantified using ROIs over metastatic bone and lymph node identified by BLI and their non-metastatic contralateral counterparts. Results were expressed as percentage of injected dose per cubic centimeter of tissue (%ID/cc).

### Ex vivo biodistribution

Animals were sacrificed at 24 h and 48 h post-injection. The following organs and tissues were collected: lymph nodes, blood, urine, kidneys, liver, spleen, pancreas, heart, lungs, brain, femur, bladder, and muscle. After resection and ex vivo scanning, organs were weighed and the counts in each organ were measured using a gamma counter (Wizard, Perkin Elmer) with correction for decay.

### Statistical analysis

Data were expressed as mean ± sd. Statistical comparisons were made using a two-tailed *t*-test using GraphPad Prism software. A *p* value of less than 0.05 was considered statistically significant.

## Results

### Synthesis and characterization of ^nat/64^Cu-MN-anti-miR10b

The synthesis of ^64^Cu-MN-anti-miR10b started with the modification of MN, a 20-nm aminated dextran-coated iron oxide nanoparticle whose synthesis was previously reported (Yoo et al. 2014). The nanoparticles have been optimized to enhance the extravasation of the agent into the interstitium of tumors and metastatic lesions (Yoo et al. 2017a). MN nanoparticles were functionalized with NODAGA, a chelating ligand, by a coupling reaction between the amino groups and the activated ester moiety of NODAGA (Scheme 1). We chose the NODAGA chelator because of its ability to rapidly form highly stable ^64^Cu complexes, essential for preventing in vivo dissociation of the radiometal and its subsequent retention in the body. The number of NODAGA chelators per nanoparticle was quantified as 13 ± 2. The ratio of Cu/nanoparticle was determined as 14 ± 1 by ICP-MS after complex formation using ^nat^CuCl_2_. Prior to in vivo studies, the nanoparticles were treated with SPDP and functionalized with anti-miR-10b antagomirs via a disulfide linkage. The number of antagomirs per nanoparticle was characterized as 7.4 ± 0.2 following previously described procedures (Yoo et al. 2014, 2015).

**Fig. 1:**
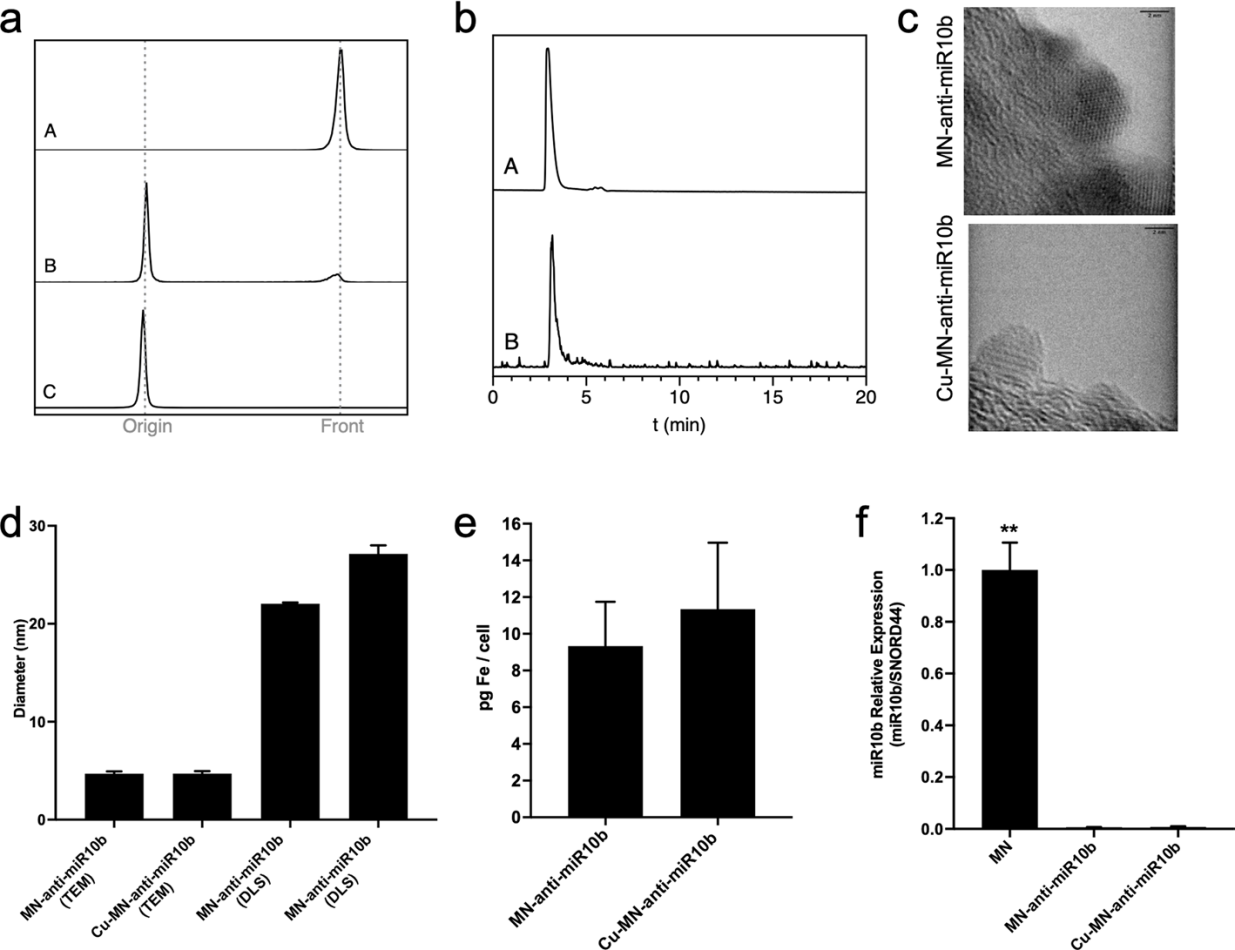
Characterization of ^nat/64^Cu-MN-anti-miR10b. **a** Radiochemical purity confirmed by iTLC. (Top) unlabeled Cu-64, (middle) separation of ^64^Cu-MN-anti-miR10b and unlabeled Cu-64, and (bottom) ^64^Cu-MN-anti-miR10b after PD-10 purification. **b** HPLC traces of ^64^Cu-MN-anti-miR10b using size exclusion chromatography, (top) UV trace at 254 nm, (Bottom) radiodetector. **c** TEM of Cu-MN-anti-miR10b and ^nat^Cu-MN-anti-miR10b. **d** Nanoparticle size by TEM and DLS. **e** In vitro cell uptake by breast adenocarcinoma cells. **f** qRT-PCR demonstrating target engagement (inhibition of miR-10b). ^nat^Cu was utilized for the preparation of ^nat^Cu-MN-anti-miR10b (*t*-test, *n* = 3, **P < 0.01)

Evaluating the in vivo biodistribution of ^64^Cu-MN-anti-miR10b requires the development of efficient labeling and purification methods. The radiolabeling of MN-anti-miR10b was achieved in acetate buffer at pH 6.8, 60 °C for 20 min. These conditions are favorable for the labeling of NODAGA chelators while maintaining the integrity of the nanocarrier. After purification on a PD-10 column, ^64^Cu-MN-anti-miR10b was obtained with a radiochemical purity > 99% (Fig. 1a) with a specific activity of 7.1 mCi/mg of iron. Radiochemical identity was confirmed by size-exclusion chromatography (Fig. 1b). In addition, ^nat^Cu-MN-anti-miR10b was synthesized to evaluate the effect of the presence of Cu-NODAGA chelates on the nanoparticle size and target engagement. The size of the iron oxide crystals in the core was measured as 4.71 ± 0.24 nm for ^nat^Cu-MN-anti-miR10b and 4.69 ± 0.23 nm for MN. The surface modifications did not cause any significant changes in the size and crystal structure of the iron oxide core as shown by transmission electron microscopy (TEM, Fig. 1c). The hydrodynamic diameter of the dextran-coated functionalized nanoparticles, ^nat^Cu-MN-anti-miR10b, was determined as 27.1 ± 0.9 nm, which is 5.1 nm larger than that of parent MN. The introduction of Cu-NODAGA and the anti-miR10b antagomir resulted in a 23% increase in hydrodynamic diameter (Fig. 1d).

The cellular uptake, expressed as elemental iron per cell was 9.33 ± 2.42 pg Fe per cell for MN-anti-miR10b and 11.34 ± 3.62 pg Fe per cell for ^nat^Cu-MN-anti-miR10b, which was not significantly different (Fig. 1e). Finally, the RNA-enriched cell extracts were analyzed to compare the inhibition of miR10b by qRT-PCR. Compared with the expression level of miR10b after treatment with parent MN devoid of antagomir, miR10b expression was completely inhibited following treatment with MN-anti-miR10b or ^nat^Cu-MN-anti-miR10b (Fig. 1f).

### Biodistribution of ^64^Cu-MN-anti-miR10b

**Fig. 2:**
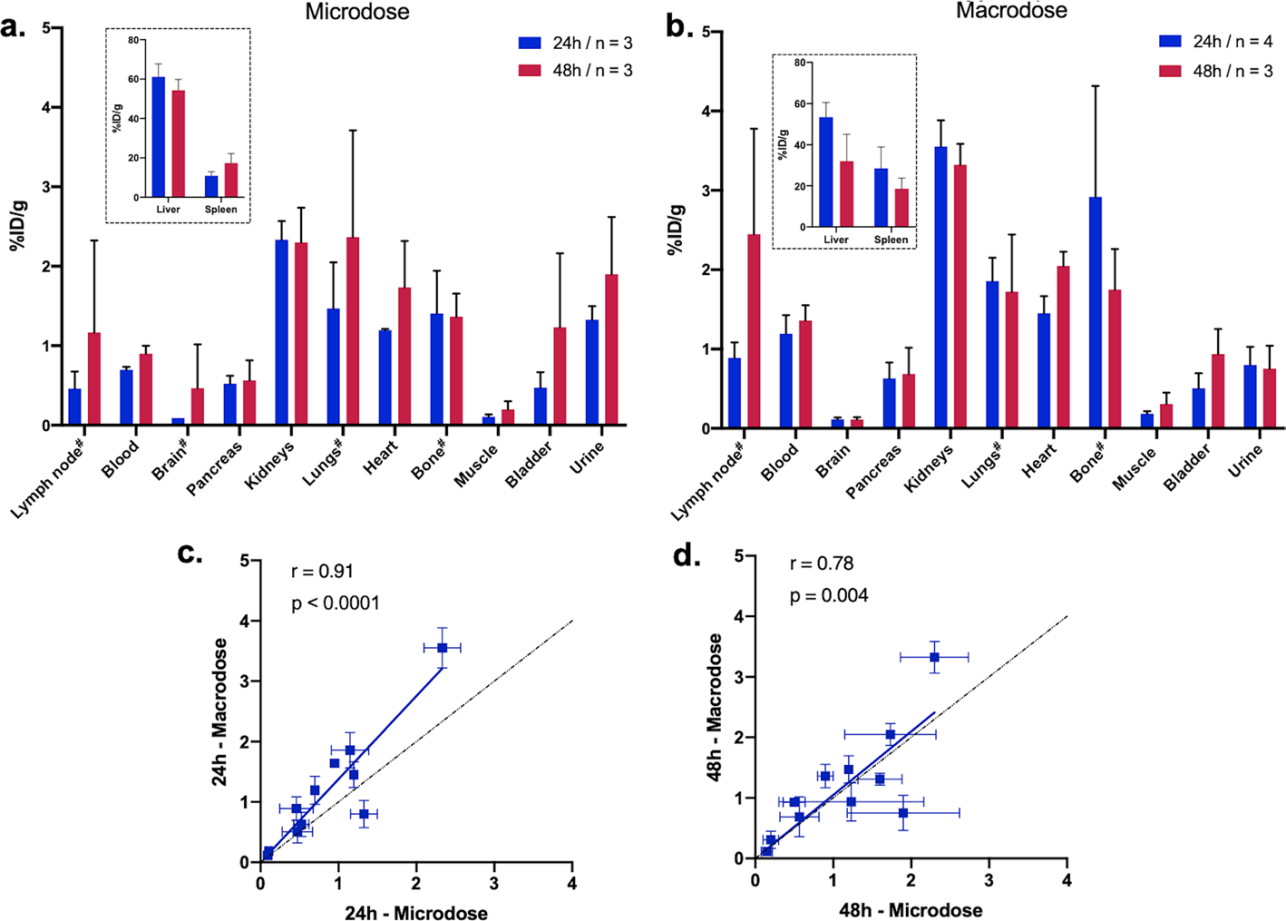
Ex vivo biodistribution measured at 24 and 48 h after administration of **a** a microdose of ^64^Cu-MN-anti-miR10b (%ID/g), **b** a therapeutic carrier-added macrodose of ^64^Cu-MN-anti-miR10b (%ID/g). Insets show the %ID/g values in liver and spleen. ^#^Denotes organs with metastasis as detected by BLI. Error bars represent standard error of the mean. Correlation between the %ID/g in non-metastatic organs obtained after administration of a microdose and the %ID/g obtained after administration of a macrodose at **c** 24 h and **d** 48 h post-injection (**c** and **d** Pearson product–moment correlation, the dashed line corresponds to the line of identity)

Next, we evaluated the biodistribution of ^64^Cu-MN-anti-miR10b by PET–MRI and ex vivo gamma counting in a total of 13 mice bearing luciferase-expressing metastatic breast adenocarcinomas (4T1-luc2). 4T1-luc2 cells express luciferase and can be detected by non-invasive bioluminescence imaging (BLI). Two different concentrations of ^64^Cu-MN-anti-miR10b were investigated: (1) a tracer-level dose of ^64^Cu-MN-anti-miR10b, referred to as microdose (1 mg Fe/kg, *n* = 6), and (2) a therapeutic dose, referred to as macrodose, corresponding to ^64^Cu-MN-anti-miR10b co-injected with MN-anti-miR10b (15 mg Fe/kg, *n* = 7). Mice were scanned by PET–MRI at different time points. After in vivo imaging, the mice were killed at 24 h p.i. (*n* = 3 in the microdose group and *n* = 4 in the macrodose group) and 48 h p.i. (*n* = 3 in each group) for ex vivo biodistribution evaluation (Fig. 2). Time-activity curves for liver, kidney and heart obtained from the PET images (Additional file 1: Figure S1) indicate that most of the injected dose is rapidly taken up by the liver. This is in line with previously reported studies (Briley-Saebo et al. 2004; Estevanato et al. 2012; Schlachter et al. 2011).

This is also supported by the ex vivo biodistribution analysis indicating that most of the injected dose was present in the liver and the spleen with %ID/g values in the liver of 61.2 ± 6.5 (24 h, microdose), 53.4 ± 7.1 (24 h, macrodose), 54.3 ± 5.5 (48 h, microdose) and 32.1 ± 13.0 (48 h, macrodose) (Fig. 2a, b, see insets). %ID/g values lower than 4 were measured in all other harvested organs and tissues. Because ^64^Cu-MN-anti-miR10b accumulates in metastatic lesions, higher %ID/g values were observed in organs bearing metastases such as lymph node, brain, bone, and lung (Fig. 2a, b and Additional file 1: Figure S2, # denotes organs with metastatic lesions). Moreover, lymph node metastases were larger in the macrodose group explaining the higher %ID/g values obtained compared to the microdose group. A comparable biodistribution was observed between the microdose and the macrodose at 24 h and 48 h post-injection (Fig. 2a, b and Additional file 1: Figure S2, note the presence of metastatic lesions in lymph node, brain, lungs, and bone). This is also reflected by the strong correlation with slope near unity that is observed between the %ID/g obtained after administration of a microdose and the %ID/g obtained after administration of a macrodose at 24 h and 48 h p.i. in non-metastatic organs, with a Pearson coefficient of 0.91 (*p* < 0.0001, slope = 1.38) and 0.78 (*p* = 0.004, slope = 1.05), respectively (Fig. 2c, d).

### PET–MRI of ^64^Cu-MN-anti-miR10b accumulation in tumors and metastases

Two weeks after orthotopic tumor cell implantation, once metastases were confirmed by BLI, the mice were injected intravenously with ^64^Cu-MN-anti-miR10b. The metastatic organs were identified by in vivo and ex vivo BLI. The uptake of ^64^Cu-MN-anti-miR10b by metastases was evaluated by PET–MRI following injection of a no-carrier added microdose (20 μg as Fe, 118–190 μCi per mouse, *n* = 6) or a carrier-added macrodose, as specified above (300 µg as Fe, 127–135 µCi per mouse, *n* = 7). After in vivo PET–MR imaging, the mice were killed at 24 h and 48 h p.i. for ex vivo PET–MR imaging.

**Fig. 3:**
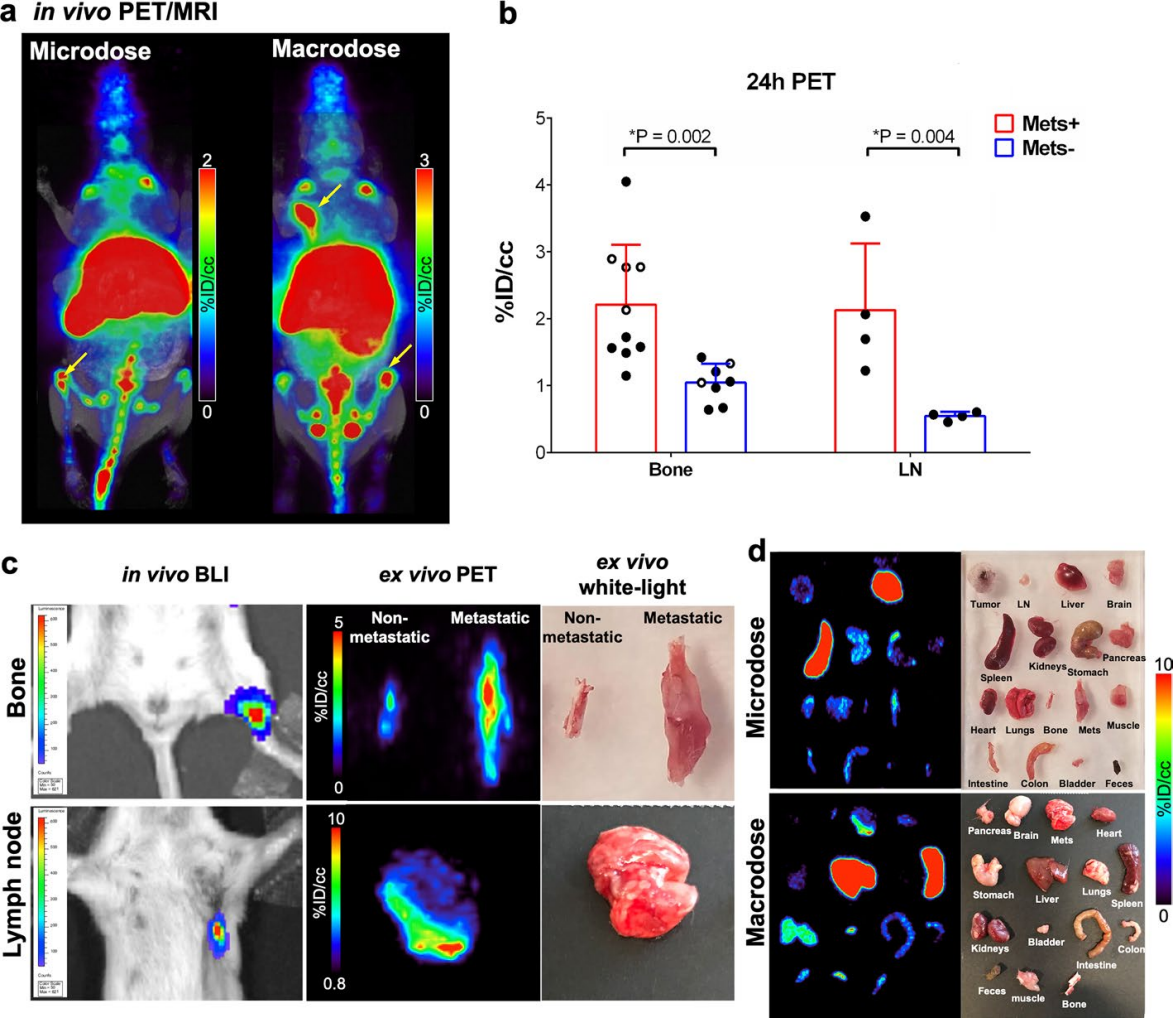
PET–MRI of ^64^Cu-MN-anti-miR10b uptake by metastatic lesions. **a** In vivo PET–MRI maximum intensity projection (MIP) images of mice bearing metastatic breast adenocarcinoma 24 h after injection of a microdose or a macrodose of ^64^Cu-MN-anti-miR10b. The yellow arrows point to bone or lymph node (LN) metastasis. **b** Quantitation of ^64^Cu-MN-anti-miR10b accumulation in metastatic (Mets+) and non-metastatic (Mets−) organs obtained from in vivo PET images at 24 h post-injection (%ID/cc). The high signal intensity in the metastatic organs compared to the non-metastatic organs confirms uptake of the therapeutic by the metastases. Open circles represent mice injected with a microdose and closed circles represent mice injected with a macrodose. **c** Ex vivo PET–MRI of bone and lymph node metastases. From left: in vivo BLI, ex vivo PET, and ex vivo white-light photograph of metastatic lesions. **d** Ex vivo biodistribution of ^64^Cu-MN-anti-miR10b as visualized by PET 48 h after microdose injection and 24 h after macrodose injection

Consistent with the results from the biodistribution studies, the liver and spleen demonstrated a very high PET signal, indicative of hepatic clearance. Renal clearance was the other major clearance pathway as shown by a high PET signal in the kidneys and urine (Fig. 2a, b). Bone and lymph node metastatic lesions could be identified by in vivo PET–MRI, partly because of their spatial separation from the liver and spleen (Fig. 3a). Time-activity curves from metastatic and non-metastatic bones after injection of a microdose of ^64^Cu-MN-anti-miR10b showed higher uptake by metastases (Additional file 1: Figure S3). At 24-h after injection of microdose or macrodose of ^64^Cu-MN-anti-miR10b, the metastatic lymph nodes and bone identified by BLI showed significantly higher %ID/cc than the corresponding organs devoid of metastases (Fig. 3b).

BLI images of bone metastatic lesions after injection of a microdose of ^64^Cu-MN-anti-miR10b are shown in Fig. 3c. The uptake of ^64^Cu-MN-anti-miR10b by metastatic lesions was further confirmed by ex vivo PET–MRI (Fig. 3c). The metastatic bone and lymph nodes could be identified by in vivo BLI. These metastatic lesions exhibited higher ex vivo PET signal than their non-metastatic counterparts.

In agreement with ex vivo biodistribution and in vivo PET–MRI, ex vivo imaging showed that the activity associated with the excised liver and spleen was highest in both the macrodosing and microdosing studies. High activity was also seen in organs colonized by tumor cells, such as the lymph nodes, bone, and lungs (Fig. 3d).

Taken together, these observations support a methodology for radiolabeling and imaging of MN-anti-miR10b and other similar nanotherapeutics. Our findings point to the feasibility of clinical PET–MRI of therapeutic accumulation in metastatic lesions, as a critical step on the path to translation of related therapeutic agents.

## Discussion

We previously identified microRNA-10b as a master regulator of the viability of metastatic tumor cells and designed the therapeutic miR-10b inhibitor, MN-anti-miR10b, for the treatment of metastatic cancer (Ma et al. 2007; Yigit et al. 2013; Yoo et al. 2015). We demonstrated that MN-anti-miR10b caused complete and persistent regression of local lymph node and distant metastases in breast cancer models with no evidence of systemic toxicity (Yoo et al. 2015, 2017b).

In this study, we performed key translational experiments that will bring us closer to applying MN-anti-miR10b in patients with advanced metastatic cancer. We developed a radio-labeled derivative of the MN-anti-miR10b therapeutic, ^64^Cu-MN-anti-miR10b. We demonstrated that radiolabeling the therapeutic did not affect its physicochemical properties, did not impact cellular uptake in vitro, and preserved effective engagement of its target, based on the complete inhibition of miR10b in tumor cells. We then showed that microdosing PET–MRI would adequately reflect the biodistribution of a therapeutic dose of the agent and be effective at highlighting metastatic tissues, based on their uptake of ^64^Cu-MN-anti-miR10b.

The tools and methods described here would allow us to demonstrate delivery of the therapeutic to clinical metastases and clarify the biodistribution of the agent in cancer patients. Indeed, one of the major challenges facing the development of similar therapeutics lies in the effective delivery to the target organs. In the case of drug delivery to metastases, complicating factors include the larger size of the lesions, as compared to animal models, the heterogeneity of human disease, and differences in the pharmacokinetics of the drugs, due to interspecies hemodynamic variability. Based on these differences, it is not possible to directly extrapolate proof of successful clinical implementation of therapeutic agents from preclinical biodistribution and efficacy data.

For that reason, the capacity to carry out microdosing PET studies in patients under an exploratory investigational new drug application protocol represents an important step on the path to clinical approval. Since the PET technique is sensitive enough to determine the concentration of radiolabeled drug with sensitivity approaching the subpicomolar range, as little as a microgram of the radiolabeled drug is generally sufficient to perform the proposed PET study in humans. This characteristic has significant advantages in the initial phases of drug development. Because the low mass of the radiolabeled drug does not induce drug effects, approval from the U.S. Food and Drug Administration for initial human studies may be obtained more quickly and with a more limited preclinical safety and toxicology dossier than is required for therapeutic agents.

Nevertheless, it is not obvious that the microdose will show a linear relationship to the biodistribution and pharmacokinetics of a therapeutic dose. Even though for intravenous compounds there is generally a direct proportionality within a twofold range (van Nuland et al. 2019), non-linearity of the relationship is possible due to saturation of absorption, distribution, metabolism, and excretion (ADME) at the high doses, and saturation of the target at the low doses. The latter reason is particularly common for monoclonal antibodies, which demonstrate a shorter half-life at low doses because of engagement with limited quantities of endogenous target antigen (Burt et al. 2020). However, in the present study, the fact that we see the same behavior between the microdose and therapeutic dose indicates that it is reasonable to move forward with a microdosing study in patients.

The impact of such an exploratory imaging study would be threefold. First it would establish that MN-anti-miR10b, which is so effective in mice, will also accumulate in human metastases. This greatly de-risks the clinical development of the therapeutic because it shows drug delivery is indeed feasible. Nanotherapeutics have failed because of poor delivery and the fact that human tumors are larger than in mice with different surface-to-volume ratios. Second, the proposed studies will reveal the pharmacokinetic behavior of MN-anti-miR10b which will allow one to establish dosing during therapy. Third, once MN-anti-miR10b reaches clinical trials, one can use the radiolabeled drug to select patients for treatment, based on which patients’ metastases accumulate the therapeutic.

The successful synthesis and testing of ^64^Cu-MN-anti-miR10b is also significant because it sets a precedent for the testing of similar nanotherapeutics based not only the iron oxide delivery platform, as illustrated here, but also on other nanoparticles that present the possibility of delivering multimodal therapy. For example, copper-based nanomedicine, such as copper cysteamine could be employed to combine RNA-based targeted therapy with copper-cysteamine based X-ray induced photodynamic therapy, which has shown promise in cancer (Li et al. 2010; Ma et al. 2014; Shrestha et al. 2019).

With specific relevance to RNA-based therapeutics, the vast majority of these agents rely on a delivery vehicle, which in many cases comprises a lipid nanoparticle (e.g., Alnylam’s Onpattro, MedImmune’s MEDI1191 and AstraZeneca’s AZD8601) or GalNAc (e.g., Alnylam’s Givlaari, Novartis’ Inclisiran, etc.). In some of these cases, it may be possible to employ a related radiolabeling and imaging protocol on the path to full clinical trials, in order to not only de-risk clinical development by demonstrating successful delivery but also to gain further insight into target engagement as a function of dose, schedule, or drug design.

## Conclusions

The value of clinical microdose imaging studies will be in answering one of the most key questions on the path to drug development without the associated cost to do a full clinical trial—the question of delivery to the target organ sites. In this study, we developed a radiolabeled MN-anti-miR10b therapeutic by introducing NODAGA and radioactive ^64^Cu. The biodistribution of ^64^Cu-MN-anti-miR10b was investigated after the injection of a microdose, which showed a comparable biodistribution to the therapeutic macrodose in all organs. We expect that the described radiolabeling and imaging protocols will advance the clinical development of similar nanotherapeutics by elucidating the pharmacokinetic behavior of the agents, de-risking future clinical trials, and assisting in the selection of patients for treatment, based on which patients’ metastases accumulate the therapeutics.

## Supplementary Information

**Additional file 1: Figure S1.** Time–activity curves obtained from PET images from 5 min to 48 h post-injection in **a** heart, **b** kidney and **c** liver from mice (*n* = 3) that were administered a microdose of ^64^Cu-MN-anti-miR10b. Error bars represent the standard deviation. **Figure S2.** Biodistribution of ^64^Cu-MN-anti-miR10b injected at a microdose and a standard therapeutic dose (macrodose) measured at **a** 24 and **b** 48 h after injection. ^#^Denotes organs with metastasis as detected by BLI. Results are expressed as %ID/g. Error bars represent the standard deviation. (*t*-test, *P < 0.05, **P < 0.01). **Figure S3.** Time-activity curves obtained from PET imaging from 5 min to 48 h post-injection of a microdose of ^64^Cu-MN-anti-miR10b in metastatic and non-metastatic bones (*n* = 3).

## Data Availability

The datasets generated during and/or analyzed during the current study are available from the corresponding authors on reasonable request.
