# Supplementary material for: Radiolabeling and PET–MRI microdosing of the experimental cancer therapeutic, MN-anti-miR10b, demonstrates delivery to metastatic lesions in a murine model of metastatic breast cancer
Source: Cancer Nanotechnol. 2021 Jul 8;12(1):16. doi: 10.1186/s12645-021-00089-5 (PMC8442631; doi:10.1186/s12645-021-00089-5)
Supplement: Supplementary file 1 — Additional file 1: Figure S1. Time–activity curves obtained from PET images from 5 min to 48 h post-injection in a heart, b kidney and c liver from mice (n = 3) that were administered a microdose of 64Cu-MN-anti-miR10b. Error bars represent the standard deviation. Figure S2. Biodistribution of 64Cu-MN-anti-miR10b injected at a microdose and a standard therapeutic dose (macrodose) measured at a 24 and b 48 h after injection. #Denotes organs with metastasis as detected by BLI. Results are expressed as %ID/g. Error bars represent the standard deviation. (t-test, *P < 0.05, **P < 0.01). Figure S3. Time-activity curves obtained from PET imaging from 5 min to 48 h post-injection of a microdose of 64Cu-MN-anti-miR10b in metastatic and non-metastatic bones (n = 3). [file 12645_2021_89_MOESM1_ESM.docx]

Supporting information for

**Radiolabeling and PET-MRI microdosing of the experimental cancer therapeutic, MN-anti-miR10b, demonstrates delivery to metastatic lesions in a murine model of metastatic breast cancer**

Mariane Le Fur^1,2^, Alana Ross^1^, Pamela Pantazopoulos^1^, Nicholas Rotile^1,2^, Iris Zhou^1,2^, Peter Caravan^1,2*^, Zdravka Medarova^1*^, Byunghee Yoo^1*^

*^1^MGH/MIT/HMS Athinoula A. Martinos Center for Biomedical Imaging, Massachusetts General Hospital and Harvard Medical School, Boston, MA 02129, USA.*

*^2^Institute for Innovation in Imaging, Massachusetts General Hospital, Boston, MA 02129, USA.*


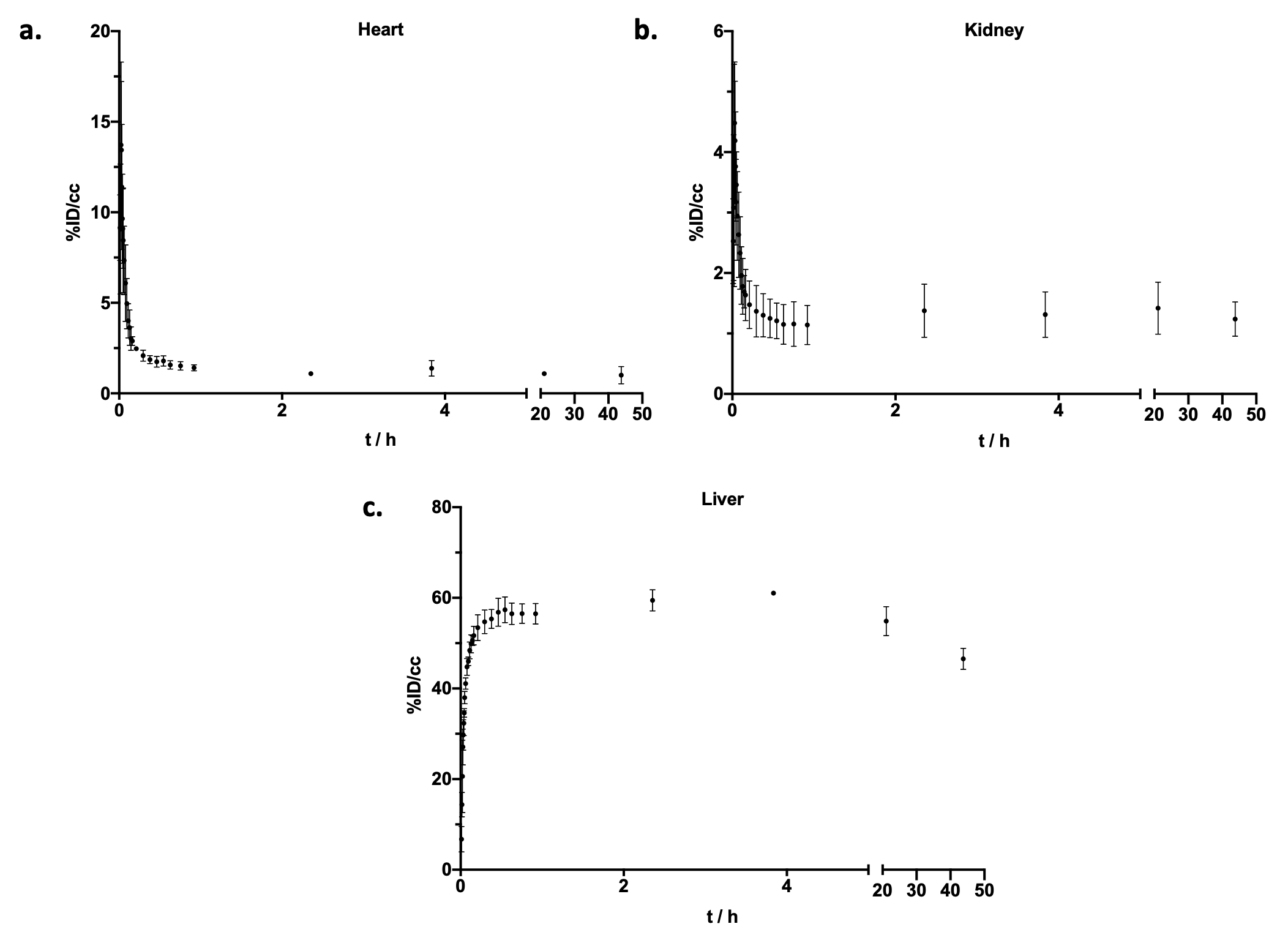


**Figure S1.** Time-activity curves obtained from PET images from 5 min to 48 hrs post injection in **a.** Heart, **b.** Kidney and **c.** Liver from mice (n = 3) that were administered a microdose of ^64^Cu-MN-anti-miR10b. Error bars represent the standard deviation.


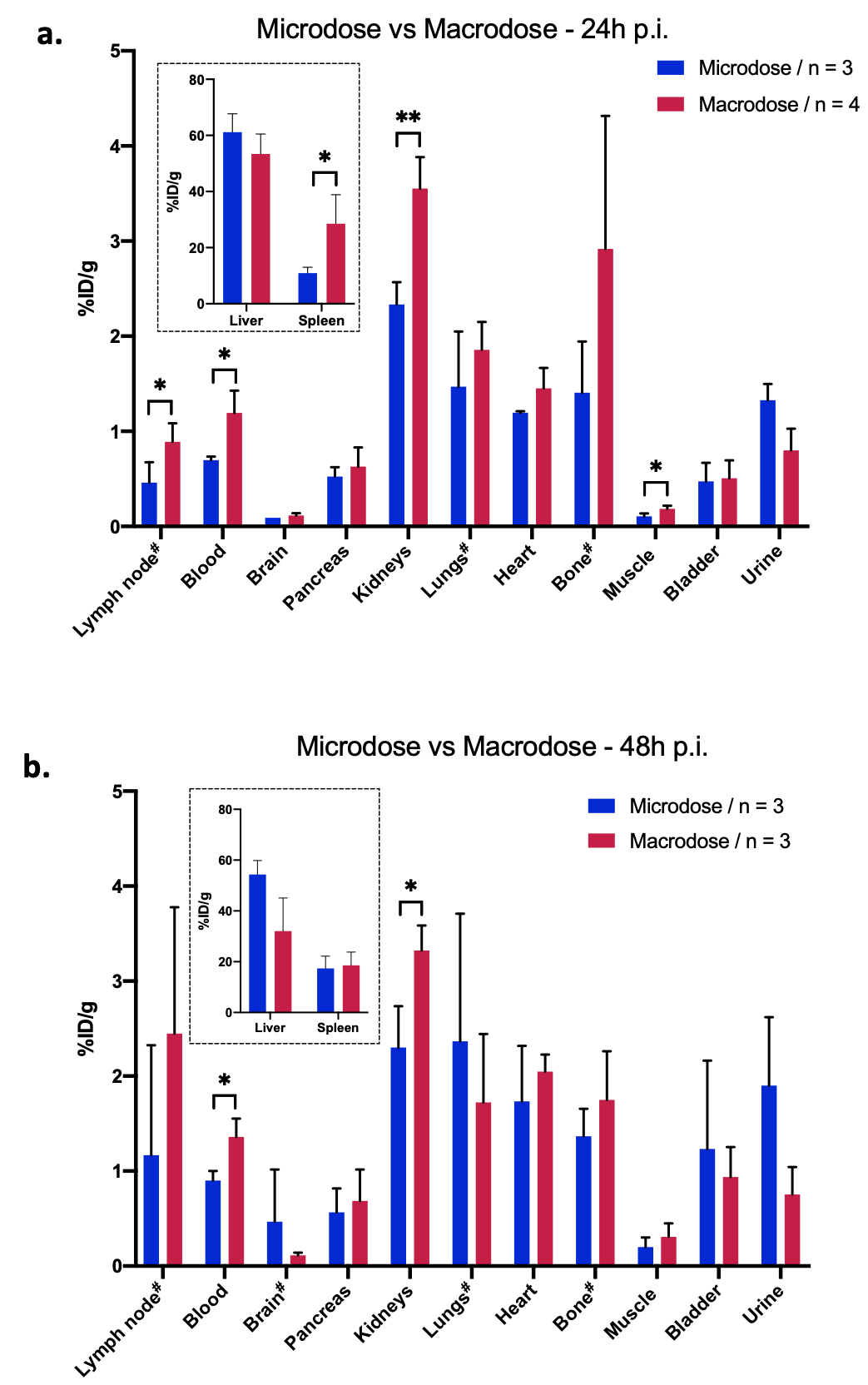


**Figure S2.** Biodistribution of ^64^Cu-MN-anti-miR10b injected at a microdose and a standard therapeutic dose (macrodose) measured at **a.** 24 and **b.** 48 hours after injection. ^#^ Denotes organs with metastasis as detected by BLI. Results are expressed as %ID/g. Error bars represent the standard deviation. (t-test, **P* < 0.05, ***P* < 0.01).


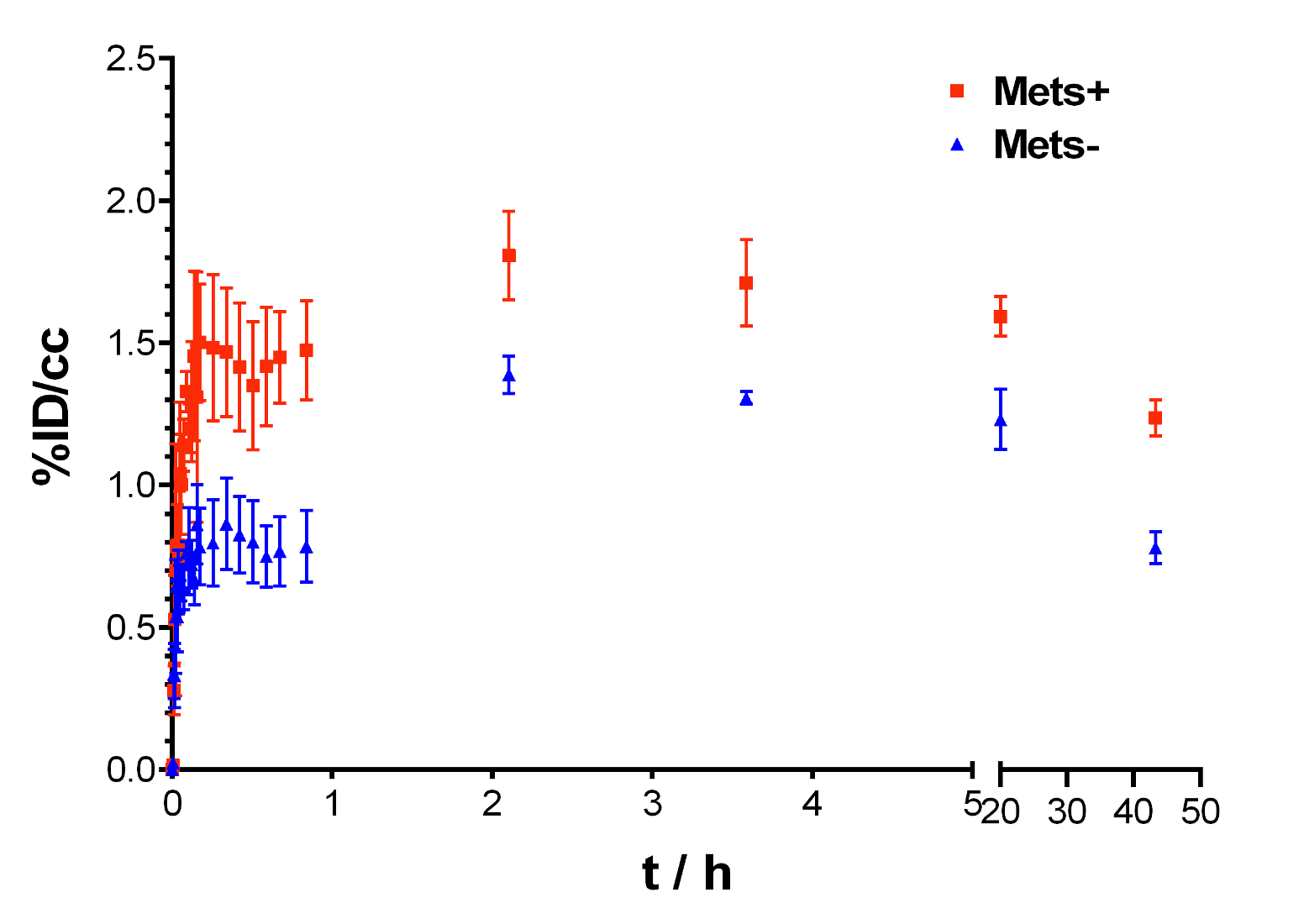


**Figure S3.** Time-activity curves obtained from PET imaging from 5 min to 48 hrs post injection of a microdose of ^64^Cu-MN-anti-miR10b in metastatic and non-metastatic bones (n = 3).
